# Supplementary material for: Are carbon emissions trading and green financial instruments synergistic? -Comprehensive quantitative research based on content analysis
Source: PLoS One. 2024 Mar 7;19(3):e0298601. doi: 10.1371/journal.pone.0298601 (PMC10919601; doi:10.1371/journal.pone.0298601)
Supplement: S1 Data — (ZIP) [file pone.0298601.s001.zip › Data/地级市Z_F工作报告.zip!/─■╧─/╥°┤¿╩╨/└┤╫╘╣1⁄2╓┌║┼í╛┬φ┐╦╩2╛▌═°í┐_2015.docx]

各位代表：

　　现在，我代表市人民政府向大会报告工作，请予审议，并请政协委员和其他列席人员提出意见。

　　一、拼搏奋进，经济社会发展迈出新步伐

　　2014年是我们积极应对经济下行压力，奋力推进改革发展取得新成绩的一年。全市上下在转型升级、优化环境、改进作风三个方面作出示范的新要求，抢抓机遇，迎难而上，坚持开放内涵式发展，较好地完成了市十四届人大二次会议确定的各项目标任务。全年实现地区生产总值1395.67亿元，同比增长9.5%，增速比全区平均水平高1.5个百分点，居五市之首；完成地方财政收入251.70亿元，增长12.7%，其中公共财政预算收入153.60亿元，增长14.1%；全社会固定资产投资1392.76亿元，增长21.2%；社会消费品零售总额382.47亿元，增长9.9%；进出口总额45亿美元，增长86.7%；城镇居民人均可支配收入26118元，增长9.1%；农村居民人均可支配收入首次突破万元大关，达到10275元，增长10%。经济社会发展呈现稳中有进、稳中提质的好势头。

　　——产业结构调整实现突破。出台《产业转型升级和结构调整实施方案》，大力推进经济工作项目化，着力“稳一优二提三”，产业结构出现新变化。工业结构优化升级。及时出台稳定工业增长、首台（套）产品认定等扶持政策，全力“保增长、保降耗”，全年完成规模以上工业增加值471.71亿元，增长10.5%。生态纺织园如意科技25万锭高档纱线、中银绒业500万件羊绒衫及210万件高端羊绒服饰，宝丰能源60万吨烯烃等107个重点项目建成投产，新增产值117亿元。轻工业占比提高4个百分点。经济技术开发区战略性新材料、数字化装备制造等创造了国际国内多个行业“单项冠军”。高新技术产业和装备制造业占规上工业比重提高1.8个百分点。银川科技园开园运营，引进中科院银川中心、浙江大学技术转移中心等研发机构、科技公共服务平台近20家，成为国家科技成果转化服务（银川）示范基地。新认定国家、自治区级企业技术中心6个。推进企业“两化”融合，加快改造传统产业，六大高耗能产业占比下降4.5个百分点，万元GDP能耗下降17.55%，扭转了“十二五”以来能耗居高不下的局面。新兴产业迅速起步。IBI育成中心软件动漫、文化创意、物联网、云计算等新兴产业加速聚集，入驻企业308家，主营业务收入达31.26亿元，网上交易额2890亿元，获得国家级知识产权试点园区和工业设计创意产业综合服务平台等称号，对我市乃至全区生产性服务业发展的示范引领作用初步显现。引进中兴集团建设大数据中心，荣获“智慧城市商业模式创新和中国领军智慧城市”大奖，入选首批“宽带中国”示范城市。世界电子竞技大赛落户并成功举办首届比赛。淘宝特色中国宁夏馆、京东商城宁夏馆等国内知名电商平台上线运营，网上商品销售快速增长，全市电子商务交易额突破32亿元。服务业提速发展。服务业投资占比提高4.6个百分点，完成增加值578.74亿元，增长7.2%。“双十工程”进展迅速，西夏万达、上陵波斯顿酒店等建成开业；公铁物流城、润恒物流园等项目加快推进，实现社会物流总额2822.67亿元，增长11%。举办房车博览会、贺兰山国际岩画峰会等展会、活动40场次，旅游接待人数和旅游收入分别增长20.67%和19.5%。引导房地产业健康发展，商品房销售面积增长18.4%。“三精”农业加快发展。完成农业总产值106.83亿元，增长6%。新建改造现代农业示范园区（基地）30个，新增设施园艺1.1万亩，建成永久性蔬菜基地3.9万亩，中地、月牙湖万头奶牛生态牧场运营良好，有机水稻、酿酒葡萄、适水产业加快发展。累计培育农业产业化龙头企业207家、家庭农场247个、农民专业合作社655家，成立全区首家优质水稻产业联合体，农产品加工转化率达到64%。特色产业占农业总产值比重达90%以上，占农民收入比重超过51%。

　　——美丽银川建设成效明显。修改完善《银川市城市总体规划（2011－2020）》，编制完成全域银川总体发展战略策划、产城一体化规划及生态规划，未来城市发展理念、产业定位、生态导向全面确立。永、贺两县与市区同城化、灵武融入开放核心区步伐加快。以安置区建设为重点的旧城（棚户区）改造加快推进，建成安置区183万平方米，回迁安置群众1.7万余户，改造老旧小区67万平方米，特色小城镇、中心村建设进展迅速，城乡一体化步伐加快。滨河、兵沟、永宁3座黄河大桥进展顺利，新建改造大连路等城市道路31条、小街巷10条；实施部分道路雨污分流改造，启动第七、第九污水处理厂建设，第八水厂建成供水，基础设施综合配套功能不断提升。全力推进主干道大整治大绿化、爱伊河景观提升等一批重点生态绿化工程，绿博园一期、11个小微公园建成向市民开放，新建完善园林绿化面积1500公顷、造林7467公顷，市域绿量扩大、档次提升。蓝天工程扎实推进，高标准完成二排沟、四二干沟等综合治理，实施工业废气治理项目21个，拆除老旧锅炉152台，淘汰黄标车、老旧车9727辆，空气质量优良天数达到274天，比2013年增加23天。完善“大城管”体制机制，在三区成立城管巡回法庭，出台《“门前三包”责任制管理办法》、《停车场规划建设管理办法》，交通秩序、户外广告、渣土车洒漏等专项整治成效明显，清理“两违”建筑993万平方米，城市精细化管理不断加强。荣获“亚洲都市景观奖”唯一城市综合奖。

　　——改革开放步伐全面加快。重点改革成果丰硕。完成市县两级政府机构改革，工商、质监、药监三局合一。实施行政审批权相对集中改革，在全国省会（首府）城市率先组建行政审批服务局，市民大厅建成运行，社会各界反响良好；开展行政审批事项“清零”规范，保留78项、减幅48%，取消免征82项行政事业性收费，为企业和群众减负2.5亿元。全年新登记注册企业9095户，增长68.8%。“金融强市”取得重大突破，成立金融工作局，组建金融控股、西部（银川）担保等4家地方金融机构，成立3家国有资本运营公司；引进金融机构6家，浦发银行银川离岸业务创新中心揭牌运营，宁夏网虫、天佳科技等11家企业在“新三板”挂牌上市；发行城投债、私募债42亿元，争取棚户区改造融资贷款184亿元。金融业增加值占第三产业比重达到23.5%。

　　农村产权制度改革稳步推进，基本完成农村集体土地确权登记发证，贺兰县在全区率先开展土地信托流转试点，永宁县探索成立粮食银行，全市累计流转土地52.81万亩，占承包耕地的51.6%。开放型经济加快发展。滨河新区“四城”建设成效显现，完成大环境绿化9万亩，建成滨河中学、横城花园一期等23个项目，国际医疗城、科教城部分项目主体封顶，全年完成投资70亿元。综合保税区注册企业87家，黄金珠宝文化产业园、懿丰葡萄酒等项目基本建成，跨境贸易电子商务出口业务上线运行，进口肉类指定口岸加快建设，获批水果种苗指定口岸，进出口贸易突破17亿美元，实现了封关运行一年见效的预期目标。临港经济区“六通一平”基本完成，建成陆港物流园一期。阅海湾中央商务区“三园一轴”基础设施建成向市民开放，国际交流中心全面启用，宁夏建筑设计院等总部楼宇落成投用，入驻企业89家，进入了运营见效新阶段。开放通道加快贯通，新开通5条国际客运航线，实现所有省会城市航线全覆盖，开辟银川至青岛港等出海通道。与瓦努阿图维拉港市等2个城市建立国际友城关系。成功举办银川首尔经贸推介会、呼包银榆经济区第一届经济技术合作洽谈会等活动，实施招商引资项目245个，到位资金466亿元，引进世界和中国500强企业7家；积极支持优势企业“走出去”，中银公司并购盛大游戏，梅花生物并购伊品生物。我市对外开放和国际化步伐加快。

　　——民生服务水平不断提高。全年民生支出192亿元，占公共财政预算支出的73%。为民办10件实事全部完成。实施青年大学生创业“扬帆计划”和电子商务创业培训计划，IBI育成中心成为国家级创业孵化示范基地。全年培养小老板2889人，培育小企业1448个，农村劳动力转移就业11.57万人，新增城镇就业6.26万人。新建扩建中小学、幼儿园30所，金凤区、西夏区通过义务教育均衡发展“国检”，兴庆区、永宁县通过“区检”，优质教育资源扩面提升，进入全国首批教育质量综合评价改革试验区。宁夏幼儿师范学校升格为高等专科院校。启动公立医院综合改革，推行市三医院由宁夏医科大附属医院托管、市中医院法人治理结构试点，推广“先住院、后付费”诊疗模式。10所社区卫生服务机构和157所标准化村卫生室投入使用，开展政府购买社区卫生服务试点。市第一人民医院与清华大学第一附属医院建立合作关系，银川卡瓦中心免费救助儿童先天性心脏病50例，国际医疗城引进7个顶尖专科。“单独两孩”政策启动实施。县（市）区文化馆、图书馆实现全覆盖，成功举办西部放歌·诗歌节、环青海湖国际公路自行车赛银川赛段、自治区第十四届运动会等大型活动，市民文化体育生活丰富多彩。鼓励被征地农民参加城镇职工基本养老保险，实行城镇职工、城乡居民大病医疗保险，提高城乡低保标准，推行殡葬惠民政策。建成市妇女儿童活动中心。公租房与廉租房合二为一，新纳入住房保障3245户5497人，住房公积金支持保障性住房建设力度加大。累计搬迁移民12744户56416人，移民地区人均可支配收入达到7540元，增长16%。加大肉菜储备投放、市场调控、价格监管力度，居民消费价格指数累计上涨2.1%。积极推进社会治理创新，兴庆区和谐社区建设试点经验在全区推广，建立食品监管新模式，打击传销、吸毒人员“大收戒”等社会治安专项行动成效显著，安全生产形势保持平稳，群众安全感、幸福感不断提高。民族团结进步创建活动扎实开展，灵武市、闽宁镇荣获全国民族团结进步模范集体。国防动员、双拥工作稳步推进，防灾减灾、地震、气象、统计、仲裁、档案、地方志等工作取得新进展。

　　——政府自身建设切实加强。扎实开展党的群众路线教育实践活动，积极转作风、抓发展，针对查摆出的“四风”突出问题和群众反映强烈的问题，市政府领导班子制定整改措施78项，已完成73项，整改率93.5%。严格执行中央八项规定及区市若干规定，全面清理规范公务用车、办公用房，会议、文件和评比表彰项目分别减少30%、29.5%、16.8%，“三公”经费下降23.8%。主动接受人大及其常委会的法律监督和工作监督，自觉接受政协的民主监督，认真听取各民主党派、工商联、无党派人士、人民团体和社会各界的意见，全年提请人大审议地方性法规4部，制定政府规章、规范性文件12件，议案建议提案办结率100%。在全区率先开展行政复议权相对集中试点。高度重视并切实抓好国家土地利用管理督察、土地出让金审计反馈问题的整改落实，进一步规范了土地管理和财政资金使用。创新电视问政、施政“三位一体”监督模式，开展“两不一慢”整治行动，问责和处理89人，机关和干部作风明显好转，政府效能和执行力进一步提高。

　　各位代表，刚刚过去的一年，在诸多困难和挑战面前，我们知难而进，顶住了压力，经住了考验，虽然增速有所放缓，但发展质量和效益明显提高，生态纺织园、综合保税区、iBi育成中心、绿博园、市民大厅建设运行等多个方面刷新了银川速度，创造了银川效率，推动经济社会实现了新发展。成绩来之不易。这是自治区党委、政府和市委正确领导的结果，是市人大、政协监督支持的结果，是全市人民艰苦奋斗、共同努力的结果。在此，我代表市人民政府，向辛勤奋战在各条战线的广大干部群众，向给予政府工作大力支持的人大代表、政协委员、各民主党派、工商联、无党派人士和人民团体，向驻银部队指战员、武警官兵、公安民警，向为银川改革发展作出贡献的社会各界朋友，表示衷心的感谢和崇高的敬意！

　　在看到成绩的同时，我们也清醒地认识到发展中还存在一些突出问题：一是经济稳定增长的压力仍然较大，投资持续增长的动力不足，新兴产业和服务业规模还不大，改革创新的力度还不够，结构调整和转型升级的任务十分艰巨。二是城乡居民收入增长还不快，保障改善民生有待加强；群体性上访、涉法涉诉、违法违规建设等影响社会和谐稳定的因素不容忽视。三是基础设施配套功能还不完善，城市地下管网老化，存在安全隐患；精细化管理还有不到位的地方，老旧小区、城乡结合部和农村环境有待提升。四是法治政府建设需要加强，个别干部依法行政水平不高，执行力不强，抓落实不够，发展环境有待进一步优化。这些问题，我们将认真对待，采取针对性措施加以解决，努力让人民群众满意。

　　二、适应新常态，全面加快开放内涵式发展

　　今年是全面深化改革的关键之年，是全面推进依法治市的开局之年，也是全面完成“十二五”规划的收官之年。市委十三届五次全会要求适应新常态，抓住新机遇，加快推进开放内涵式发展。新常态下，国家不断优化空间发展布局，继续推进西部大开发战略，加大对中西部地区承接产业转移、基础设施建设等方面的倾斜支持，将为我们加快新型工业化、城镇化，持续扩大投资和消费需求带来更多政策利好；国家重点实施“一带一路”倡议，自治区提出加快宁东基地、综合保税区、滨河新区、生态纺织园、空港物流园区深度融合发展，争取成立国家级经济开发新区，这是我们加快建设试验区核心区、丝绸之路经济带战略支点的重大机遇。当前我市经济总体向好，率先调整转型的效果初步显现，轻纺、新材料、信息产业、电子商务等新产业、新业态正在形成，开放载体不断夯实，改革红利不断释放，对经济持续健康发展的支撑作用进一步增强。我们要坚定信心，主动作为，保持平稳较快发展势头，不断创造新优势，培育新增长点，开创银川科学发展、跨越发展新局面。

　　今年政府工作总的要求是：认真贯彻落实党的十八大和十八届三中、四中全会，以及中央经济工作会议和自治区党委十一届五次全会、自治区“两会”、市委十三届五次全会精神，坚持稳中求进工作总基调，抢抓“一带一路”、“两区”建设等历史机遇，以提高经济发展质量和效益为中心，深入实施“2258”工作思路，突出深化改革和扩大开放，突出转型升级和创新发展，突出美丽银川建设，突出改善民生，突出依法治市，加快开放内涵式发展和国际化现代化进程，全面完成“十二五”规划目标任务，推动开放、富裕、和谐、美丽银川建设取得新成效。

　　经济社会发展的主要预期目标是：地区生产总值增长10%左右，地方财政公共预算收入增长12%左右，全社会固定资产投资增长22%左右，社会消费品零售总额增长10%，外贸进出口总额增长15%，城镇居民、农村居民人均可支配收入均增长10%，出生人口政策符合率达到95%以上，居民消费价格指数控制在3.5％以内，城镇登记失业率控制在4％以内，完成自治区下达的节能减排指标任务。

　　各位代表，确定这样的目标，既是我们高点定位，发挥示范引领作用的责任使命，也是我们自加压力、起跳跨越的现实需要。实现这样的目标，必须坚持加快发展不动摇，直面挑战不退缩，全力以赴抓产业，千方百计扩总量，改革创新求突破，为稳增长、调结构、促转型、惠民生注入持续动力。扎实开展“项目建设年”活动，建设一批、投产一批、储备一批，形成重大项目梯次推进、持续发力的格局，力争全年完成投资1700亿元。进一步优化投资结构，加大新兴产业、基础设施配套、生态建设、民生和公共服务领域投资力度，提高政府投资效率，扩大企业和社会投资。科学编制“十三五”规划，争取更多大项目进入国家和自治区计划盘子。创新招商引资理念和方式，着力引进国内外知名企业和战略投资者，力争全年引进到位项目资金增长10%以上。我们必须目光放远，步伐稳健，政策更新，措施更实，全面超额完成目标任务，推动开放内涵式发展行稳致远。

　　三、坚持创新驱动，大力推进产业转型升级

　　坚持市场导向，加大产业结构调整力度，力促经济发展由要素、投资驱动向创新驱动转化，推动经济结构由工业主导型向服务业主导型转变。

　　加速新型工业化。制定实施新兴产业发展规划，坚持做大优质增量与调整优化存量“两手抓”，推进工业化、信息化深度融合，打造西北地区战略性新兴产业发展示范区。规模以上工业增加值增长11%左右。提升园区承载力。建立政府主导、企业为主、市场化运作机制，完善园区电力、供水、污水处理等基础设施建设。健全差异化、特色化考核评价机制，发挥好经济技术开发区产业转型升级引领带动作用，促进生态纺织园与宁东基地、银川科技园与iBi育成中心等园区联动发展，提升产业配套水平和集聚效应。加快西夏区老工业基地调整改造步伐，出台相关配套政策，重点实施以产业发展为基础、财政增收为核心的“西夏突破”工程。开展闲置低效用地清理行动，提高园区土地利用率和产出效益。加快“轻、高、新”产业发展。推进生态纺织园“一园多区”投产运营和达产达效，力促如意科技5000万米色织布、3000万件衬衣，中银绒业220万米高端精纺面料，综保区10万头气流纺高档纱线等项目建成投产，加快培育和引进上下游关联（配套）企业，打通纺织工业全产业链。大力发展智能制造、新材料产业，实施奥钢联高端铸件、天通及佳晶蓝宝石等重点项目，启动建设晶体材料产业园，引进加工企业，打造全球最大的单晶硅生产基地和国内最大的工业蓝宝石生产基地。加快发展通航产业，启动建设通航产业园及西北地区小飞机交付中心，举办西部航空展及跳伞节，发展飞机制造、维修、飞行培训、航空服务等相关产业。加速发展信息产业，抓好中兴（银川）大数据中心、iBi育成中心二期项目，办好世界电子竞技大赛和微电影节，力争iBi育成中心主营业务收入突破40亿元，网上交易额达到4000亿元，加快物联网、大数据、云计算、游戏动漫软件等新兴产业集聚发展，全面推进智慧银川和信息消费试点城市建设。加大企业培育扶持力度。支持如意、中银、宝塔、宝丰等龙头企业走兼并重组、资本运作的路子，进一步提高品牌知名度和产品附加值，推动产业向价值链的高端、向“微笑曲线”的两端发展。实施“个转企、小升规”工程，引导中小微企业为优势产业和大企业协作配套，发展“专精特新”中小企业10家，新增规上企业40户。推动落实差别电价、电力直接交易等优惠政策，创新小微企业“服务超市”、银政企对接等服务平台，降低企业发展成本。引导企业加快技术改造，实施西北轴承智能化热处理、伊品色氨酸等60个技改项目，淘汰落后产能56万吨，促进冶金建材、生物医药等传统产业优化升级。抓好城市矿产示范基地、银峰铝业循环经济产业园等项目建设，推进产业节能化、循环化发展。

　　推动服务业提质提速。抓好国家服务业综合改革试点，统筹发展生产性和生活性服务业，打造现代服务业示范区，不断提高服务业的质量和水平，服务业增加值增长8%以上。建设区域性物流中心。积极引进国内外知名物流、快递企业，争取菜鸟公司智能物流骨干网节点落户银川，抓好公铁物流城、润恒二期、苏宁云商等重点项目建设，大力发展大宗商品物流、冷链物流和第三方物流，提高城市配送和物流信息化水平，物流业增加值增长6.5％以上。推进电子商务示范城市建设。实施宁港新经济产业服务园、宁夏电商谷等项目，建好海尔（银川）虚实网服务园、曹操购物网等本土电商平台，打造区域性电子商务产业聚集区和电子商务培训基地，促进消费向生产和经营领域延伸。打造西部国际旅游目的地。整合提升东西两条文化旅游带功能，加快推进三沙源、华夏河图·生态小镇、军博园二期等项目，打造贺兰山东麓葡萄文化长廊。抓好西夏陵申遗及西夏博物馆迁建，完成韩美林艺术馆、规划展示馆布展运营，新建银川文化创意基地，推动文化旅游融合发展。加大宣传推介，开拓韩国、台湾的入境游市场。完善旅游接待服务体系，推动观光游向休闲度假游转变，让游客住下来、能消费，旅游总收入增长10%以上。促进商贸服务业提档升级。推进永泰城、新华联广场、建发大阅城、亘元万豪等“双十工程”建设，完善“一主两副”商圈功能，打造特色街区和社区商业生活服务圈。培育发展健康及养老服务业。顺应消费结构升级和人口老龄化趋势，出台促进健康服务业发展的实施意见，引导支持社会资本投向医疗保健、体育健身、健康养老等领域，推进银川爱心护理院、法国爱德府养生养老等项目建设，完善健康服务体系，打造区域健康养老服务中心。

　　加快农业现代化。以“三精”农业为抓手，以农业提质增效、农民持续增收为核心，促进一二三产业融合发展，加快转变农业发展方式，打造国家现代农业示范区和农产品质量安全区，农业总产值增长5%以上。发展壮大特色农业。重点发展五大优势特色产业，推进设施园艺、有机水稻、奶畜产业、适水产业、酿酒葡萄规模化、标准化、专业化生产，新建改造农业科技示范园区（基地）30个，建设蔬菜标准园区10个、标准化养殖示范场35个以上、大水面生态养殖示范点10个，新增酿酒葡萄1.2万亩。培育新型经营主体。引进、扶持一批龙头企业、流通主体，完善“种养加销”全产业链，培育国家、自治区级“合作示范社”和“星级家庭农场”60家，组建水产联合体，农产品加工转化率达到68%。健全农产品质量安全体系，完成绿色、有机农产品认证10个。加强农产品冷链体系建设，完善营销网络，让更多的优质特色农产品打入国内外市场。提高农业综合效益。建设高标准农田45万亩，新增高效节水灌溉面积5万亩。实行最严格的土地管理制度，依法保护耕地、基本农田和水源地，治理农村面源污染。鼓励农业科技人员下乡入户、创新创业，加大新品种、新技术、新装备推广应用，加强农业气象、信息化等社会化服务体系建设，培养职业农民1.5万人次，农业科技贡献率达到67%，主要农作物综合机械化作业率达到91%。

　　突出创新引领发展。完善科技创新体系。提升经济技术开发区、高新技术产业开发区创新带动能力，完善科技园等科技产业园区功能，加快建设研发中试基地和高新技术产业孵化基地，打造国家科技创新试验示范区。突出企业创新主体地位，完善企业科技创新后补助机制，引导企业加大科技投入，新培育高新技术企业10家、科技型中小企业50家。加快科技成果转化。对接优势特色产业发展需求，支持搭建产学研协同创新平台，实施各类科技项目100个，使更多的科技成果就地转移转化，变成实实在在的产业和产品。加强知识产权运用和保护，为科技创新装上“安全阀、保护器”。推进人才管理改革试验区建设。实施人才优先发展战略，组建人才服务联盟、西北人才金港等平台，大力引进和培养高层次科技人才、高水平创新团队。鼓励支持企业通过技术入股、股权激励等方式引进人才，吸引科技人员创新创业。

　　四、深化改革开放，不断增强发展动力活力

　　按照自治区“改革落实年”的要求，围绕发展面临的突出问题推进改革，围绕构建开放型经济新格局扩大开放，打造“两优”环境高地。

　　加大改革力度。继续简政放权。加快建立政府部门权力清单、责任清单和企业投资项目负面清单，进一步厘清政府权责边界，激发市场和社会活力。稳步推进事业单位分类改革，扩大法人治理结构试点。再取消下放一批行政审批事项，对保留的事项进行动态管理并及时向社会公布。加强市民大厅及行政审批服务局运行制度体系建设，同步推进网上办事和电子监察，打造现代化的政务服务平台，实现“一局一章管审批、一个窗口办成事”。深化财政体制改革。把今年定为“财源建设年”，坚持“工业富市”与“金融强市”并重，千方百计做大财力总量。把政府性债务纳入预算管理。设立产业发展基金，探索建立财政投入基金化、政府投资资本化制度，放大财政资金效应。建立统一的政府购买服务平台和机制，制定购买服务目录清单；推行政府和社会资本合作（PPP）等模式，支持社会资本进入市政基础设施、公益事业等领域。完善金融服务体系。引进华夏、光大银行等金融机构，筹建寿险公司、融资租赁公司、股权投资服务中心等地方金融机构，稳步发展P2P、众筹、第三方支付等互联网金融，打造区域金融中心。支持企业从资本市场直接融资，力争培育20家中小企业在“新三板”挂牌上市。加快企业征信体系建设和运用，推进诚信银川建设。加快国有企业改革。推进以管资本为主的国有资产监管体制改革，建立完善市属国有企业经营预算、绩效考核和收益上缴制度。组建通联资本、创新资本、新兴产业资本运营公司，发展混合所有制经济，吸引社会资本参与新兴产业和现代服务业发展。抓好农村综合改革。全面完成农村集体土地所有权、承包地经营权、宅基地使用权及房屋所有权确权登记颁证。探索建立城乡统一的建设用地市场。发展多种形式的适度规模经营，建立农村产权交易中心，推广土地信托流转、粮食银行、农机作业公司等模式，稳步推进农村集体资产产权制度改革，壮大村集体经济。出台户籍制度改革配套政策，实行统一的居住证制度，让进城农民同等享受城市基本公共服务。

　　加快试验区核心区建设。推进阅海湾中央商务区和滨河新区、综合保税区、临港经济区“三位一体”开放核心区建设，打造对阿合作和总部经济新高地。加快阅海湾中央商务区运营见效。抓好绿地中心、阅海金融大厦等重点项目，促进庆华集团、工行宁夏分行等企业总部建成或入驻，努力实现“双百”目标。出台扶持奖励政策，发展会展经济，集聚园区商气人气。高标准建设滨河新区。坚持新型产城一体化、“四城”融合发展之路，加快完善基础设施和生态环境大框架。突出核心功能区建设和产业支撑，着力推动国际科教城、国际医疗城、滨河产业园、横城整体开发实现突破，加快通用航空、大数据、医疗旅游、休闲度假等新兴产业和高端服务业聚集发展，建设宜居宜业宜游的生态田园城。推动综合保税区转型发展。培育壮大黄金珠宝、葡萄酒等主导产业，力争引进1~2家世界或中国500强企业，全年进出口总额达到20亿美元以上。拓展完善口岸功能，高标准建设进口肉类、进境水果种苗口岸，积极发展融资租赁等业务，开展黄金珠宝、红酒及进出口商品保税展示交易。完成配套生活区和二期建设。积极对接上海等自贸区发展，抓好海关监管、检验检疫等贸易便利化的创新制度落地，试点国际贸易单一窗口管理模式，争取离港退税等政策，推进“三个一”便利化通关，争设国际邮件交换站。促进临港经济区融合发展。加快与综保区、滨河新区、宁东基地联动发展，承接发展航空物流、出口加工等外向型产业。

　　构建开放大通道。重点打造空中、陆上、网上丝绸之路，努力在对阿、对东北亚等国家开放合作上实现新突破。协调推进银川河东国际机场三期扩建，开通银川至迪拜等国际客货运航线，规划建设银川国际航空物流中心，发展航空物流和临空经济。积极布局中欧班列运行节点城市，开拓陆路经中亚直达欧洲的货运班列，加强与天津、青岛、深圳、郑州及霍尔果斯、二连浩特等重要口岸的通关协作，构建多式联运、向西出境、向东出海的国际物流大通道。推进迪拜杰贝阿里自贸区与银川跨境电商平台互联互通。

　　五、统筹城乡发展，加速新型城镇化进程

　　落实自治区空间发展战略规划定位，突出核心带动，加快建设大银川都市区，打造生态宜居、富有活力、独具特色的国际化现代化城市，争创联合国人居奖。

　　提升城乡规划建设水平。优化城市空间和功能布局，调整完善土地利用总体规划，促进“三规合一”、“多规融合”。编制实施总体城市设计、特色风貌研究和建筑导则。加快永宁、贺兰与主城区“三个同步”、灵武与开放核心区深度融合发展，严格执行“三低一绿”标准，保持舒朗、大气、清爽的城市风格。完善基础设施配套，开展地下综合管廊试点。启动第九水厂、西夏区与金凤区给水管道连通工程，实施城市供热主管网建设，加快老旧天然气管网改造，确保城市供水供气安全。推进四座污水处理厂扩容提标改造，启动贺兰山东麓山洪沟等治理工程，健全防洪排水体系，提高城市综合承载能力。加快棚户区改造，完成42个631.35万平方米安置区建设，确保群众按期回迁，年内基本消除城中村。实施老旧小区改造50万平方米，建好镇北堡、良田等7个特色小城镇、15个美丽乡村，大幅提升农村人居和生态环境。

　　加快构建综合交通体系。协调推进银西高铁建设，启动城际轨道等立体交通体系规划。推动滨河、兵沟、永宁黄河大桥局部建成通车，实施青银高速银川段改扩建，规划109国道永宁至贺兰段东移迁线工程，新建延伸六盘山路、宝湖路、沈阳路等道路20条，改造小街巷10条，完善“东西贯通、南北相连”的路网格局。加快公交优先发展，启动BRT二号线及火车站综合客运交通枢纽设计建设，优化线网布局和站点设置，提高公交运营服务水平和出行分担率，创建公交示范都市。加快建设步行、自行车等慢行系统，促进绿色低碳出行。完善智能交通系统，优化交通组织管理，改造拥堵节点，挖掘现有道路通行能力，合理布局停车设施，解决好市区局部交通拥堵和“停车难”问题。

　　优化生态环境。持续做好“绿化、水系、空气”三篇文章，把“碧水蓝天、明媚银川”的城市品牌擦得更亮。巩固提升主干道大整治大绿化成果，重点实施110国道综合整治、正源北街、贺兰山路等道路景观提升工程，抓好绿博园二期、城南运动休闲生态园等项目，新建10个小微公园，提高绿化和园林景观品位。加快治理银新干沟等城市“龙须沟”，抓好爱伊河水生态环境整治、水洞沟水土流失治理等项目，实施华雁湖、鸣翠湖、鹤泉湖等湖泊湿地清淤治理和恢复工程。开展“空气质量专项会战”，加强空气污染源监测解析和综合施策，加大燃煤锅炉整治力度，实施多热源联网集中供热工程，推广清洁高效供热方式，抓好工业废气、机动车尾气、建筑扬尘治理，让银川的水更绿、天更蓝、空气更清新，让老百姓生活在生态田园、美丽的银川。

　　促进城市管理提档升级。全面提升城市精细化、人性化、智能化管理水平，营造文明优美宜居的城市环境。实施城乡一体化洁净工程，突出抓好无物业老旧小区、城乡结合部、市区沟渠环境卫生，有效整治乱停乱放、黑车营运、占道经营，加强门头牌匾、户外广告、建筑工地及渣土车规范化管理。完善环卫基础设施，提高机械化清扫率和保洁水平，推进环卫作业专业化、市场化改革。创建国家生活垃圾分类收集示范城市。推进智慧城市综合管理，促进跨区域、跨部门、跨行业信息共享和业务协同，建设覆盖全市的电子政务网络和公共服务云平台，实现城市信息化管理、民生精细化服务。深化文明城市建设，继续评树重奖“最美银川人”，抓好市民文明素质养成，培育城市人文精神，促进文明城市向城市文明提升。

　　六、突出改善民生，着力提高公共服务水平

　　经济发展的新常态更要保持民生工作的“超常态”。要进一步加大民生投入，优先解决民生问题，继续为民办好10件重点实事，让城乡居民的幸福指数进一步提高。

　　加大民生工作力度。以创业带动就业。完善创业政策和服务体系，组建创业大学，启用人力资源市场，培育打造创业孵化示范基地，提高创业成功率。落实好加快非公经济发展的政策措施，稳定中小微企业就业，多渠道开发就业岗位，重点做好高校毕业生、城镇困难人员、农村转移劳动力就业工作，新增城镇就业4.5万人。努力让每一个人都有人生出彩的机会，都能实现创业创富的梦想。大力推进扶贫开发。高标准打造闽宁镇扶贫开发样板，突出“一区（县）一策、精准扶贫”，加快整村整乡推进，抓好产业发展、移民增收、设施配套、管理服务，做大做实村级互助资金，全面完成“十二五”生态移民、劳务移民安置任务，力争移民地区人均可支配收入增长15%。着力增加居民收入。进一步提高企业退休职工、居民基础养老金及优抚对象抚恤和生活补助，建立城乡低保标准自然调整及“同城化”机制。推进农民工用工实名制和工资银行直接支付制度，严厉打击恶意欠薪、拒不支付农民工劳动报酬的违法行为。提高农村教师和乡镇医护人员绩效工资水平。落实工资福利改革政策，稳步提高机关事业单位职工的工资收入。加快社会保障提标扩面。建立“五险合一”经办管理体制，推行全民参保登记和扩面征缴，全面实行失地农民养老保险制度。稳步推进机关事业单位养老保险制度改革。提高基本医保财政补助标准，实行按病种分值结算，控制医疗费用不合理增长。完善社会救助体系，加强大病医疗救助和残疾人等特殊群体扶助关爱，不能让群众因病返贫、因灾致贫。继续扩大住房保障范围，探索推行政府回购商品房用于保障性住房新模式，加强公租房公平分配和保障房配套设施建设，实现住房困难家庭应保尽保。促进房地产业平稳健康发展。抓好物价稳控。加强社区鲜活农产品公益性网点配套建设，推行“农超对接、订单直销”模式，扩大本地农产品基地直采和流通比例，启动蔬菜价格政策性保险。通过政府调节和市场调控“两只手”，保障农民的“钱袋子”和市民的“菜蓝子”。

　　加快发展社会事业。努力办好人民满意的教育。实施二期学前教育行动计划，推动学前教育向社会购买服务，有效缓解入园难、入园贵问题。稳步推进优质教育扩面提升工程，优化教育布局，加快中小学标准化建设，扩大优质高中资源覆盖面，率先在全区实现义务教育均衡发展全覆盖。创新职业教育办学机制，对接产业需求，优化专业设置，加快培养合格劳动者和技能型人才。加快特殊教育发展，优化民办教育发展环境。加强师德建设，完善教师聘用、培训、评价考核机制，促进城乡校长、教师交流，努力让每一个孩子都能接受公平、高质量的教育。着力提高医疗卫生服务水平。稳步推进市属公立医院综合改革试点，建立现代医院管理制度和财政补偿新机制，逐步取消“药品加成”。推动优质医疗资源向基层流动，推行分级诊疗和医师多点执业，提升基层医疗服务水平。启动市妇幼保健院儿童医院项目，鼓励支持社会办医。放大卡瓦中心效应，推进国际医疗合作。推动人口长期均衡发展。深入实施文化惠民工程。完善公共文化服务体系，规划建设中心图书馆，建成文化馆新馆、儿童剧院，广泛开展“文化银川·花开四季”、“书香银川·银川书香”等群众性文化活动。加强文艺精品的创排和市场推广，切实保护好非物质文化遗产。承办第八届全区少数民族传统体育运动会，举办丝绸之路国际马术大会等大型赛事活动。完善公共健身设施，打造运动休闲特色品牌，让市民快乐体育，健康生活。

　　创新社会治理。全面推进网格化管理，加强社区标准化建设，提高社区工作者待遇，增强基层组织治理能力。抓好特殊人群和网络空间的服务管理。全面完成“六五普法”，扩大法律援助覆盖面。落实涉法涉诉信访依法终结制度，改进“三调联动”机制，着力化解社会矛盾纠纷和信访积案。完善市场监管体制机制，健全食品药品安全可追溯监管体系，营造安全放心的消费环境。构建立体化治安防控体系，严厉打击侵财、涉毒等各类违法犯罪活动，深化平安银川建设。全面落实安全生产责任制，加大隐患排查治理力度，促进安全生产形势持续稳定好转。高度重视做好防灾减灾、应急管理工作。积极创建双拥模范城“八连冠”，加强国防动员、民兵预备役建设，推动军民深度融合发展。依法管理宗教事务，做好城市民族工作，争创全国民族团结进步模范市。

　　七、推进依法行政，加快法治政府建设

　　全面推进依法治市对政府依法行政提出了新要求。我们要牢固树立法治理念，运用法治方式，不断提升政府工作法治化水平，努力建设职能科学、权责法定、执法严明、公开公正、廉洁高效、守法诚信的法治政府。

　　加强科学决策。制定《重大行政决策规则》，建立公众参与、专家论证、风险评估、合法性审查、集体讨论决定的依法科学民主决策机制，使政府的各项决策于法有据，更加符合民意。建立重大决策终身责任追究制度及责任倒查机制。设立政府法律顾问团，使法律顾问在政府重大决策和推进依法行政中发挥积极作用。完善政府立法机制，加强规范性文件审查备案，切实提高政府立法质量。

　　坚持严格执法。加快行政执法体制改革，推进执法重心下移、力量下沉，加强城市管理、市场监管、公共卫生、安全生产等重点领域综合执法，试点环境保护跨部门综合执法，解决权责交叉、多头执法、执法不作为等问题。落实行政执法人员资格管理和执法责任制，细化量化行政裁量标准，建成行政处罚电子监察和“两法衔接”平台，严格规范执法行为，努力使公平正义的阳光照到每一个人的身上。

　　强化权力监督。自觉接受人大的法律监督、工作监督、政协的民主监督，深化电视网络问政和施政，完善“三位一体”常态化监督体系，放大监督效应。充分发挥工会、共青团、妇联等群团组织的重要作用。加强政府内部层级监督、行政监察、审计监督和效能督查。加大政务公开力度，重点推进财政预算决算、公共资源交易、重大项目及民生资金等领域信息公开，及时回应社会关切，接受人民群众的监督。

　　推进作风建设。认真落实“八项规定”精神，持续整治“四风”问题。严格执行党风廉政建设“一岗双责”，出台政府重大投资建设项目管理办法，抓好工程建设、土地出让、招投标、政府采购等重点领域和关键岗位监管，真正把权力关进制度的“笼子”。健全厉行节约反对浪费制度体系，坚决控制一般性支出，保持财政供养人员、“三公经费”只减不增。启动公车改革。深入开展“转作风、抓发展”活动，加大行政问责力度。严查“三不”案件，持续整治“两不一慢”行为，努力打造一支勤政为民、崇尚法治、清正廉洁的公务员队伍。

　　各位代表，新常态、新发展、新作为。让我们在自治区党委、政府和市委的坚强领导下，凝聚全市人民的智慧和力量，抢抓机遇，乘势而上，不断开创开放内涵式发展新局面，为率先建成全面小康社会、实现跨越式发展而奋斗！
